# Steroid hormones in Alzheimer’s disease: a molecular analysis of blood and cerebrospinal fluid

**DOI:** 10.1186/s13195-026-02117-1

**Published:** 2026-07-15

**Authors:** Tik Muk, Asger Wretlind, Kourosh Hooshmand, Marc Clos-Garcia, Yue Liu, Anja Hviid Simonsen, Laura M. Winchester, Tarunveer S. Ahluwalia, Petroula Proitsi, Riccardo E. Marioni, Thomas Kümler, Steen Gregers Hasselbalch, Cristina Legido-Quigley, Alzheimer’s Disease Neuroimaging Initiative

**Affiliations:** 1 Steno Diabetes Center Copenhagen, Herlev, Denmark; 2 Institute of Pharmaceutical Sciences, King’s College London, 150 Stamford Street, London, SE1 9NH UK; 3 Centre for Preventive Neurology, Wolfson Institute of Population Health, Queen Mary University of London, London, UK; 4 Danish Dementia Research Centre, Copenhagen University, Hospital, Rigshospitalet, Copenhagen, Denmark; 5 Department of Psychiatry, University of Oxford, Oxford, UK; 6 The Bioinformatics Center, Department of Biology, University of Copenhagen, Copenhagen, Denmark; 7 Department of Science and Environment, Roskilde University, Roskilde, Denmark; 8 Institute of Genetics and Cancer, University of Edinburgh, Edinburgh, UK; 9 Institute for Clinical Medicine, Copenhagen University, Copenhagen, Denmark

**Keywords:** Alzheimer’s disease, Gender difference, Steroid hormones, Cortisol, Aldosterone, Progesterone, Mass spectrometry, Vascular dementia

## Abstract

**Background:**

Alzheimer's disease (AD) disproportionately affects women, yet existing studies have been limited to single biofluids, individual hormones, or single dementia subtypes, leaving sex-stratified profiles across the cognitive impairment spectrum poorly defined. Here we address this gap by simultaneously quantifying nine steroid hormones spanning glucocorticoid (cortisol, 11-deoxycortisol), mineralocorticoid (aldosterone), progestogen (progesterone, 17-hydroxyprogesterone), androgen (testosterone, dihydrotestosterone), and estrogen (estradiol, estrone) pathways in paired CSF and plasma across five cognitive categories: no cognitive impairment, mild cognitive impairment (MCI) with AD pathology, MCI without AD pathology, AD dementia, and vascular dementia.

**Methods:**

Steroid hormones were quantified by liquid chromatography–tandem mass spectrometry in paired cerebrospinal fluid (CSF) and plasma samples drawn from the same individuals from a cross-sectional study including 204 participants across five cognitive categories at the Danish Dementia Research Centre. Gender-stratified generalized linear models adjusted for age were applied. Cortisol findings were validated externally using the Alzheimer's Disease Neuroimaging Initiative (ADNI) cohort (n = 426).

**Results:**

Female participants with AD exhibited elevated CSF cortisol (fold change [FC] = 1.13; *P* = .04) and CSF 11-deoxycortisol (FC = 1.01; *P* = .03), alongside reduced plasma progesterone (FC = 0.90; *P* = .04). Male participants with AD showed elevated plasma aldosterone (FC = 1.19; *P* = 2.81e-03; q = 0.02). CSF cortisol correlated with amyloid-β42 and phosphorylated tau in female participants. ADNI validation confirmed elevated plasma cortisol in AD, with a larger effect in female participants.

**Conclusions:**

Gender-dependent steroid hormone dysregulation in dementia highlights cortisol and aldosterone as potentially modifiable targets warranting further investigation.

**Supplementary Information:**

The online version contains supplementary material available at https://doi.org/10.1186/s13195-026-02117-1.

## Background

Alzheimer's disease (AD) is a debilitating neurological condition and the primary cause of dementia, with a significant gender gap projected to widen further over the coming decades [1]. Epidemiological studies have shown that women are approximately one and a half times more likely to develop AD compared to men, particularly above the age of 80 [2, 3]. One potential factor contributing to the difference in AD risk between men and women is their differing exposure to hormones [4].

The relationship between endogenous sex hormone exposure, in the form of the natural, cumulative presence of estrogens (including estrone, estradiol, and estriol), progesterone and testosterone, and dementia risk in women have shown inconclusive findings. Some studies have found that reproductive events related to shorter endogenous sex hormone exposure, such as early menopause, are associated with higher risk of all-cause dementia [5, 6] and poorer cognitive outcomes [7, 8]. Further studies from the UK Biobank demonstrated that women experiencing early or surgical menopause, thereby curtailing their natural hormone exposure, showed a heightened risk of all-cause dementia, whereas prolonged exposure appeared protective, particularly among those without the apolipoprotein E ε4 (*APOE* ε4) allele [9, 10]. However, contradictory evidence exists, with studies reporting that longer endogenous hormone exposure was associated with accelerated brain aging [11], amyloid-β42 (Aβ−42) and phosphorylated tau (P-tau) levels [12].

In men, lower levels of plasma testosterone have been consistently associated with all-cause dementia and AD [13–15]. Cortisol, the primary glucocorticoid produced by the adrenal cortex in response to stress, and aldosterone, the principal mineralocorticoid regulating sodium retention and blood pressure, have both been implicated in AD pathology. Plasma and cerebrospinal fluid (CSF) cortisol levels are elevated in both mild cognitive impairment (MCI) and dementia due to AD compared to healthy controls [16, 17], and high cortisol levels are associated with cognitive decline and higher AD risk measured in CSF, plasma and urine [18–20]. For aldosterone, evidence is more limited. However, one study found higher plasma aldosterone concentration associated with impaired cognitive function [21], while another study investigating individuals with primary aldosteronism revealed an increased risk for dementia in this population [22].

Collectively, these clinical studies suggest a role for steroid hormones in cognitive decline and dementia risk. To further characterize the relationship between hormonal regulation and dementia pathology, we simultaneously quantified nine steroid hormones: cortisol, 11-deoxycortisol, aldosterone, progesterone, 17-hydroxyprogesterone, testosterone, dihydrotestosterone, estradiol, and estrone, in paired CSF and plasma samples from individuals spanning five cognitive categories: no cognitive impairment, MCI with AD pathology, MCI without AD pathology, AD dementia, and vascular dementia. Paired CSF and plasma measurements were used to assess complementary aspects of steroid hormone biology, with plasma reflecting peripheral endocrine regulation and CSF more closely representing the central nervous system hormonal environment. Measuring both compartments allowed us to assess whether hormone alterations associated with cognitive impairment and dementia were primarily central, peripheral, or detectable in both compartments. Importantly, this paired approach enabled within-individual comparison of CSF and plasma hormone profiles, reducing variability related to cohort composition and allowing us to assess whether peripheral hormone levels reflect central steroid alterations. By applying gender-stratified analyses, we aimed to identify hormone profiles specific to each dementia subtype and to determine whether hormonal alterations differ between male and female participants.

## Methods

### Participants

This study included 205 participants recruited from the Danish Dementia Research Centre at Copenhagen University Hospital, Rigshospitalet, following written informed consent and in accordance with the Declaration of Helsinki. Participants were diagnosed after comprehensive evaluation [23] and categorized into five distinct dementia subgroups defined by their clinical syndrome and etiological diagnosis. All participants had measures for CSF peptide biomarkers Aβ−42, P-tau and total tau (T-tau), as part of the diagnostic routine in short CSF Aβ−42 levels were measured with a sandwich ELISA (INNOTEST® β-AMYLOID(_1–42_), Fujirebio). T-tau and P-tau in the CSF were quantified using sandwich ELISAs—INNOTEST® hTau Ag and INNOTEST® PHOSPHO-TAU(_181P_), respectively. The groups were: participants with no cognitive impairment (NCI; individuals presenting with subjective cognitive concerns but showing no objective impairment on formal assessment) (*n* = 33), participants with MCI and no AD pathology: MCI non-AD (*n* = 38), participants with MCI-AD (*n* = 21), participants with AD dementia (*n* = 81), and participants with vascular dementia (VaD) (*n* = 32). Plasma and CSF samples were stored at −80 °C until analyzed. One participant from the no cognitive impairment group was identified as an outlier and excluded due to abnormally high plasma concentrations of progesterone and 17-hydroxyprogesterone, resulting in a total of 204 participants.

### Targeted hormone panel

Steroid hormones were extracted from plasma and CSF using the same method, a full description of this steroid quantification method can be found in the supplementary material section S1. In short, 50 μL of each sample were extracted using acetone:acetonitrile (1:1 v/v) protein precipitation, followed by centrifugation, solvent evaporation, and reconstitution in methanol:water (1:1 v/v). Hormone analysis was performed using liquid chromatography-tandem mass spectrometry (LC–MS) on an Agilent 1290 UHPLC system coupled to an Agilent 6460 triple quadrupole mass spectrometer. Chromatographic separation was achieved using a Waters HSS T3 column (1.8 μm, 2.1 × 100 mm) using a binary gradient system: water containing 2 mM ammonium fluoride (mobile phase A) and methanol as mobile phase B. Detection was performed using electrospray ionization in positive mode with dynamic multiple reaction monitoring (dMRM). Each hormone was quantified using external calibration curves with specific standards (0.00075–1000 ppb, 21 points). Data processing and peak integration were performed using Agilent MassHunter Quantitative Analysis Software (version 10.2).

### Statistical analyses

All statistical analyses and data visualizations were conducted using R Studio software (version 4.3.0; Boston, MA, USA). Demographic and clinical characteristics were re-analyzed in the subset of participants included in the steroid hormone assessment as part of this secondary analysis. Data distribution was evaluated prior to the statistical analysis using the Shapiro–Wilk test for normality and Breusch–Pagan for homoscedasticity. Non-normally distributed variables were log₂-transformed prior to analysis. Group comparisons were conducted by comparing every cognitive impairment category to a prespecified reference group (no cognitive impairment, NCI), using linear models with treatment-versus-control contrasts and Dunnett adjustment for multiple-testing. Analyses were performed using the Rpackages emmeans (v1.11.0) for marginal means and contrasts, sandwich (v3.1.0) for HC3 covariance estimation, lmtest (v0.9.40) for Breusch–Pagan testing, and tidyverse for data structuring and management.

Steroid hormone measures with more than 30% missing values across samples were excluded. Principal component analysis (PCA), variable importance in projection (VIP) score analysis, and partial least squares–discriminant analysis (PLS-DA) were applied to evaluate potential group separations across cognitive impairment categories.

Hormone concentrations were log₂-transformed before model fitting. Group differences in individual steroid hormones were examined both within each gender and in the combined genders using generalized linear models (GLMs). Models were adjusted for relevant covariates, including age at visit, gender and the interaction between group and gender (gender was included only for combined-gender analyses). Fold changes (2^β) with 95% confidence intervals (CIs) and corresponding p-values were visualized using forest plots generated with ggplot2 (v3.5.1). The concentrations of steroid hormones were summarized as mean with standard error of the mean (SEM). A p-value < 0.05 was considered statistically significant. Correction for multiple testing was carried out with FDR adjustment.

Associations between steroid hormones and Alzheimer’s disease–related characteristics were assessed using Spearman’s rank correlation, with and without gender stratification. Correlation coefficients and corresponding p-values were visualized in a Z-score–normalized heatmap generated using the corrplot package in R. Given the cross-sectional design and gender-stratified subgroup sizes, post-hoc power calculations were performed for the two primary findings using the pwr package in R. Cohen's d was calculated from log₂-transformed group means and pooled standard deviations, and power was estimated using a two-sided two-sample t-test with α = 0.05.

### Cortisol validation

Data from the Alzheimer’s Disease Neuroimaging Initiative (ADNI) [24] was used as an external validation of measures of cortisol. The ADNI study was conducted in accordance with the Good Clinical Practice guidelines, the Declaration of Helsinki, and U.S. 21 CFR Part 50 (Protection of Human Subjects). Written informed consent was obtained from all ADNI participants or their authorized representatives. Full details of ADNI ethical approvals are documented at https://adni.loni.usc.edu/. A total of n = 426 participants where included, 286 categorized as cognitively healthy and 140 individuals had AD. Participants were classified according to ADNI diagnostic criteria. Cognitively normal (CN) individuals had no memory complaints, MMSE scores of 24–30, a CDR global score of 0 (with memory box score of 0), and education-adjusted scores within normal limits on the Wechsler Logical Memory II delayed recall. AD participants had MMSE scores of 20–26, a CDR of 0.5 or 1.0, and met the NINCDS/ADRDA criteria for probable Alzheimer's disease. CSF Aβ−42, T-tau and P-tau were measured using the INNO-BIA AlzBio3 multiplex immunoassay (Fujirebio, Ghent, Belgium) on the xMAP Luminex platform (Luminex Corp, Austin, TX) at the University of Pennsylvania ADNI Biomarker Core. AD was assessed by neurologic examinations and standardized neuropsychological assessments. Cortisol was measured in plasma with a targeted LC–MS platform, using a Shimadzu LC system coupled to a SCIEX QTRAP mass spectrometer. Separation was achieved on an Acquity UPLC BEH C18 column (Waters). Comparison between levels of cortisol in cognitively healthy and individuals with AD was carried out with t-tests on log-transformed normalized data.

## Results

### Participant characteristics

This study examined hormone levels in plasma and CSF from 204 participants with varying degrees of cognitive decline. The overall mean age was 69 ± 0.7 years. Participants with no cognitive impairment were youngest, mean age 65 ± 1.8 years, while those with VaD were oldest with a mean age 75 ± 1.1 years. The MCI AD, MCI Non-AD, and AD groups had mean ages of 66 ± 1.6, 68 ± 1.9, and 70 ± 0.9 years, respectively. Approximately half the participants were female (50.5%, *n* = 103), though gender distribution varied across groups. The group with the highest percent of female participants was the AD group which included 63% (*n* = 51), while the group with the lowest percent of female participants were the MCI Non-AD group had 26.3% (*n* = 10) (Supplementary Table 1). The overall mean age of female participants was 69 ± 1.0 compared to male participants with a mean age of 70 ± 0.9 (Table 1).

**Table 1:** Participant characteristics by female and male

			**Female**			
Variable	**Overall**	**NCI**	**MCI Non-AD**	**MCI AD**	**AD**	**VaD**
	*n = 103*	*n = 18*	*n = 10*	*n = 9*	*n = 51*	***n = 15***
Age	68.88 ± 0.97	66.11 ± 2.68	62.10 ± 3.44 {0.63}	67.11 ± 2.86 {0.98}	69.53 ± 1.21 {0.47}	75.60 ± 2.07 {0.02}
BMI	24.29 ± 0.44	25.07 ± 1.02	28.41 ± 2.12 {0.15}	23.52 ± 1.37 {0.75}	23.24 ± 0.53 {0.34}	24.71 ± 0.82 {0.99}
Statins [Statins used]	31 (30.39%)	6 (33.33%)	3 (30%) {1.00}	2 (22.22%) {0.67}	10 (20%) {0.31}	10 (66.67%) {0.08}
HRT [HRT used]	13 (12.75%)	3 (16.67%)	2 (20%) {1.00}	3 (33.33%) {0.37}	3 (6%) {0.36}	2 (13.33%) {1.00}
Aβ−42 (pg/ml)	616.17 ± 29.95	873.00 ± 67.25	1075.50 ± 104.49 {0.23}	448.67 ± 26.07 {2.26e-06}	454.04 ± 11.26 {2.49e-10}	653.48 ± 91.86 {2.69e-03}
P-tau (pg/ml)	62.28 ± 3.09	40.37 ± 2.87	41.50 ± 3.11 {1.00}	48.67 ± 6.90 {0.99}	71.79 ± 4.58 {0.99}	78.23 ± 9.02 {1.00}
T-tau (pg/ml)	504.83 ± 28.20	246.56 ± 20.91	274.70 ± 28.07 {0.86}	342.11 ± 50.34 {0.39}	618.40 ± 37.99 {2.41e-09}	679.68 ± 75.69 {1.28e-07}
MMSE Score	24.62 ± 0.44	27.48 ± 0.82	26.54 ± 0.65 {0.90}	27.56 ± 0.47 {1.00}	23.11 ± 0.67 {6.65e-04}	23.27 ± 1.02 {0.01}
Plasma 11-Deoxycortisol	0.80 ± 0.07	0.86 ± 0.18	0.93 ± 0.40 {1.00}	0.79 ± 0.19 {1.00}	0.80 ± 0.08 {1.00}	0.63 ± 0.07 {0.91}
Plasma 17-Hydroxyprogesterone	0.68 ± 0.03	0.74 ± 0.10	0.74 ± 0.15 {0.83}	0.67 ± 0.03 {0.84}	0.67 ± 0.03 {0.86}	0.58 ± 0.04 {0.98}
Plasma Aldosterone	0.84 ± 0.07	0.73 ± 0.07	0.73 ± 0.07 {0.97}	0.72 ± 0.06 {0.98}	0.98 ± 0.12 {0.17}	0.63 ± 0.05 {0.92}
Plasma Cortisol	72.41 ± 3.09	70.95 ± 8.71	66.21 ± 12.73 {1.00}	78.27 ± 9.56 {0.55}	74.55 ± 4.24 {0.47}	67.53 ± 6.41 {0.88}
Plasma Dihydrotestosterone	0.04 ± 0.02	0.04 ± 0.04	0.22 ± 0.16 {0.25}	0.00 ± 0.00 {0.88}	0.03 ± 0.02 {0.98}	0.00 ± 0.00 {0.83}
Plasma Estrone	0.87 ± 0.08	0.89 ± 0.16	1.31 ± 0.22 {0.54}	0.74 ± 0.31 {0.62}	0.84 ± 0.12 {0.90}	0.75 ± 0.17 {0.96}
Plasma Progesterone	0.22 ± 5.19e-03	0.24 ± 0.02	0.24 ± 0.03 {0.95}	0.21 ± 2.65e-03 {0.35}	0.22 ± 4.12e-03 {0.31}	0.21 ± 1.54e-03 {0.15}
Plasma Testosterone	1.11 ± 0.02	1.12 ± 0.06	1.16 ± 0.03 {0.83}	1.13 ± 0.03 {0.94}	1.08 ± 0.03 {0.92}	1.13 ± 0.05 {0.98}
CSF 11-Deoxycortisol	0.18 ± 1.19e-03	0.18 ± 1.94e-03	0.19 ± 3.92e-03 {0.55}	0.18 ± 2.82e-03 {1.00}	0.19 ± 1.91e-03 {0.06}	0.18 ± 2.49e-03 {0.98}
CSF Aldosterone	0.26 ± 2.80e-03	0.25 ± 7.61e-03	0.24 ± 6.07e-03 {0.94}	0.25 ± 7.29e-03 {0.88}	0.26 ± 3.89e-03 {0.22}	0.25 ± 8.04e-03 {1.00}
CSF Cortisol	3.55 ± 0.10	2.97 ± 0.22	2.90 ± 0.27 {0.99}	3.13 ± 0.18 {0.75}	3.84 ± 0.14 {6.96e-03}	3.93 ± 0.33 {0.07}
CSF Estradiol	1.93 ± 0.06	1.87 ± 0.14	1.90 ± 0.16 {0.98}	1.98 ± 0.24 {0.97}	2.06 ± 0.09 {0.59}	1.58 ± 0.09 {0.50}
CSF Testosterone	0.09 ± 7.54e-04	0.09 ± 1.52e-03	0.10 ± 8.03e-04 {0.17}	0.09 ± 2.08e-03 {0.96}	0.09 ± 1.28e-03 {0.90}	0.09 ± 1.44e-03 {0.96}

			**Male**			
Variable	**Overall**	**NCI**	**MCI Non-AD**	**MCI AD**	**AD**	**VaD**
	*n = 101*	*n = 14*	*n = 28*	*n = 12*	*n = 30*	*n = 17*
Age	70.08 ± 0.91	64.29 ± 2.50	70.61 ± 2.09 {0.09}	65.58 ± 1.95 {0.96}	71.10 ± 1.43 {0.06}	75.35 ± 1.20 {2.20e-03}
BMI	26.17 ± 0.45	26.96 ± 0.86	26.81 ± 0.98 {1.00}	25.68 ± 1.34 {0.84}	25.10 ± 0.80 {0.51}	26.99 ± 1.14 {1.00}
Statins [Statins used]	32 (33.33%)	4 (28.57%)	8 (29.63%) {1.00}	3 (25%) {1.00}	10 (34.48%) {1.00}	7 (50%) {0.42}
HRT [HRT used]	2 (2.11%)	1 (7.69%)	1 (3.7%) {1.00}	0 (0%) {1.00}	0 (0%) {0.32}	0 (0%) {0.48}
Aβ−42 (pg/ml)	618.19 ± 31.25	1005.57 ± 65.43	821.76 ± 56.07 {0.12}	505.67 ± 72.65 {4.12e-06}	405.50 ± 15.54 {3.76e-10}	418.67 ± 35.94 {1.69e-09}
P-tau (pg/ml)	52.57 ± 2.60	39.00 ± 3.25	42.73 ± 3.44 {0.96}	78.58 ± 8.02 {0.58}	60.03 ± 5.61 {0.99}	48.41 ± 4.70 {0.97}
T-tau (pg/ml)	391.27 ± 25.14	230.43 ± 21.67	305.90 ± 40.75 {0.99}	524.67 ± 64.83 {0.51}	518.13 ± 54.39 {0.44}	346.32 ± 47.58 {0.97}
MMSE Score	25.19 ± 0.39	28.41 ± 0.58	26.04 ± 0.63 {0.12}	27.17 ± 0.67 {0.73}	23.64 ± 0.65 {1.53e-04}	22.49 ± 1.10 {2.26e-05}
Plasma 11-Deoxycortisol	0.77 ± 0.07	0.93 ± 0.21	0.69 ± 0.10 {0.51}	1.29 ± 0.39 {0.89}	0.63 ± 0.07 {0.40}	0.63 ± 0.07 {0.61}
Plasma 17-Hydroxyprogesterone	0.93 ± 0.03	0.90 ± 0.06	0.88 ± 0.05 {0.95}	1.15 ± 0.15 {0.17}	0.92 ± 0.03 {0.98}	0.91 ± 0.07 {1.00}
Plasma Aldosterone	0.87 ± 0.10	0.65 ± 0.06	0.67 ± 0.05 {1.00}	0.73 ± 0.08 {0.88}	1.32 ± 0.31 {0.01}	0.69 ± 0.04 {0.93}
Plasma Cortisol	65.55 ± 2.53	69.15 ± 4.91	61.61 ± 5.12 {0.59}	74.84 ± 9.11 {1.00}	65.05 ± 4.88 {0.69}	63.39 ± 5.07 {0.90}
Plasma Dihydrotestosterone	0.70 ± 0.03	0.64 ± 0.09	0.66 ± 0.06 {0.98}	0.70 ± 0.10 {0.97}	0.73 ± 0.05 {0.86}	0.75 ± 0.07 {0.84}
Plasma Estrone	0.82 ± 0.07	0.59 ± 0.20	0.57 ± 0.13 {1.00}	0.89 ± 0.20 {0.52}	0.98 ± 0.12 {0.17}	1.11 ± 0.21 {0.17}
Plasma Progesterone	0.22 ± 2.58e-03	0.22 ± 5.61e-03	0.22 ± 5.81e-03 {0.98}	0.24 ± 0.01 {0.33}	0.22 ± 1.89e-03 {0.97}	0.22 ± 2.56e-03 {0.92}
Plasma Testosterone	3.00 ± 0.11	2.79 ± 0.27	2.85 ± 0.24 {1.00}	2.98 ± 0.40 {0.99}	3.14 ± 0.20 {0.76}	3.22 ± 0.25 {0.64}
CSF 11-Deoxycortisol	0.19 ± 1.45e-03	0.18 ± 2.38e-03	0.19 ± 3.75e-03 {0.79}	0.18 ± 3.36e-03 {0.94}	0.18 ± 1.99e-03 {0.99}	0.19 ± 3.76e-03 {0.88}
CSF Aldosterone	0.26 ± 3.68e-03	0.27 ± 0.01	0.25 ± 4.74e-03 {0.25}	0.24 ± 7.35e-03 {0.16}	0.27 ± 8.13e-03 {1.00}	0.25 ± 4.42e-03 {0.71}
CSF Cortisol	3.45 ± 0.11	3.08 ± 0.24	3.75 ± 0.28 {0.44}	2.83 ± 0.16 {0.96}	3.40 ± 0.18 {0.90}	3.78 ± 0.17 {0.24}
CSF Estradiol	2.06 ± 0.07	2.11 ± 0.20	2.03 ± 0.13 {1.00}	2.14 ± 0.20 {0.99}	2.10 ± 0.12 {0.99}	1.94 ± 0.19 {0.85}
CSF Testosterone	0.12 ± 1.42e-03	0.12 ± 3.26e-03	0.12 ± 3.69e-03 {0.95}	0.11 ± 2.45e-03 {0.96}	0.11 ± 2.63e-03 {0.94}	0.12 ± 1.77e-03 {0.98}

Values are mean ± SEM for continuous and n (%) for categorical variables respectively. {*P*-values} from linear models with Dunnett adjustment for multiple comparisons against NCI. Log_2_-transformed values used for model fitting of CSF biomarkers and hormones

### Hormone characteristics

We quantified nine unique steroid hormones across plasma and CSF samples. Eight hormones were quantified in plasma, while five were quantified in CSF. Four hormones: aldosterone, 11-deoxycortisol, cortisol, and testosterone, were successfully measured in both sample types, whereas estradiol was only detectable in CSF. Hormone concentrations spanned a 100-fold range. CSF Testosterone showed the lowest mean concentration at 0.12 ng/ml in NCI male participants and 0.09 ng/ml for NCI, while plasma cortisol had the highest mean concentration at 69.15 ng/ml NCI male participants and 74.52 ng/ml NCI female participants. Several hormones exhibited differing concentrations between the genders, notably dihydrotestosterone being detectable solely in the plasma of male participants. Hormone concentrations for each gender and cognitive group are provided in Table 1.

### Hormones associated with cognitive impairment subtypes

**Fig. 1:**
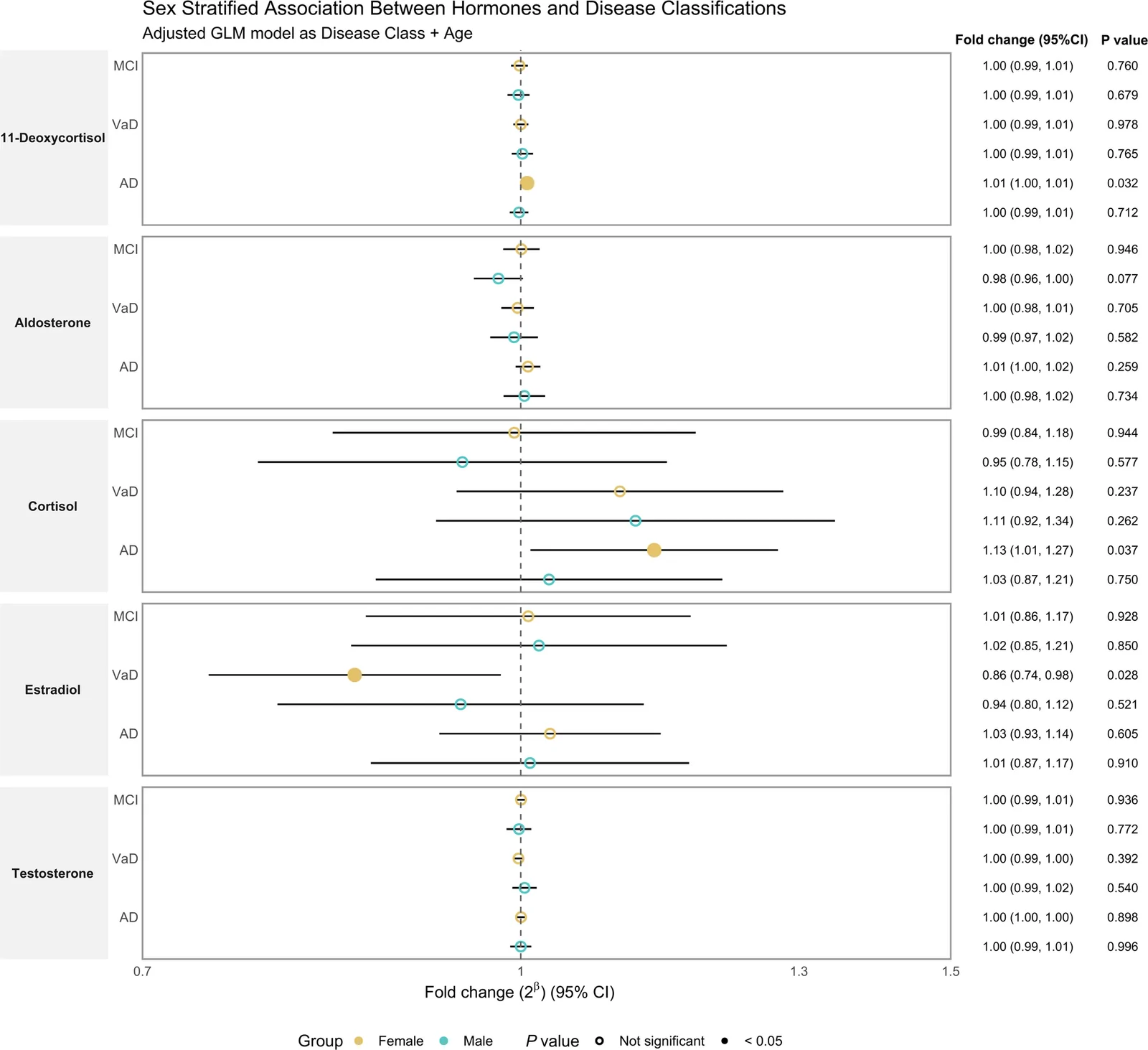
CSF gender stratified GLM. Forest plot of association between CSF hormones and disease classification using GLM. Hormone concentrations were log₂-transformed before model fitting. Fold change with 95% confidence interval against NCI and *p*-values are shown on the right

Gender-stratified GLMs adjusted for age revealed distinct hormonal patterns in CSF and plasma across diagnostic groups. Female participants with AD exhibited significantly elevated levels of CSF 11-deoxycortisol and CSF cortisol (Fold change (FC) (95% CI) = 1.01 (1.00–1.01), p-value = 0.03 and FC (95% CI) = 1.13 (1.01–1.27), p-value = 0.04, respectively) (Fig. 1, 3A-B), while female participants with VaD showed decreased CSF estradiol levels (FC (95% CI) = 0.86 (0.74–0.98), *p*-value = 0.03) (Fig. 1,Table 1). No significant associations between CSF hormones and cognitive impairment were observed in male participants.

**Fig. 2:**
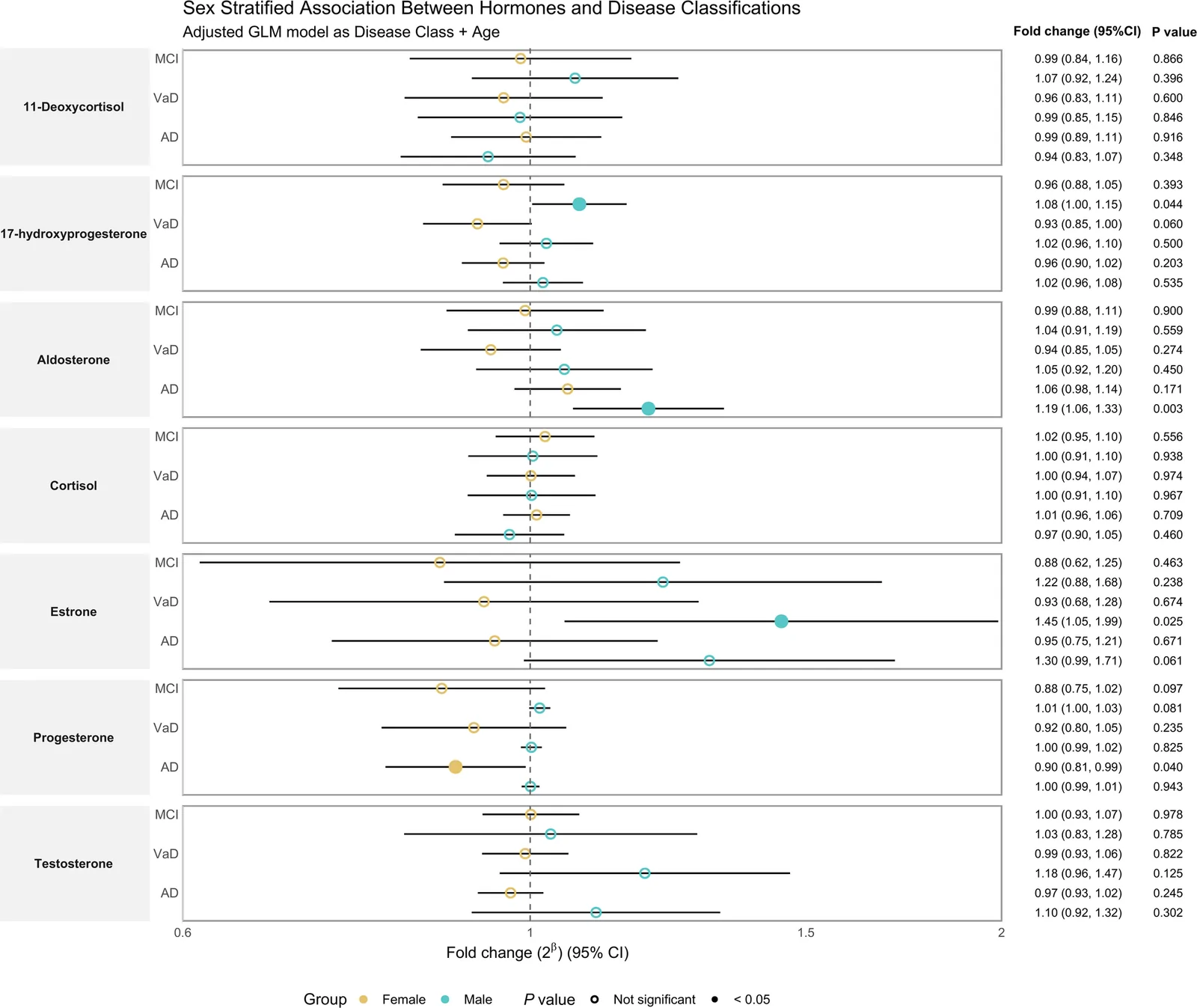
Plasma Gender stratified GLM. Forest plot of association between plasma hormones and disease classification using GLM. Hormone concentrations were log₂-transformed before model fitting. Fold change with 95% confidence interval against NCI and p-values are shown on the right

**Fig. 3:**
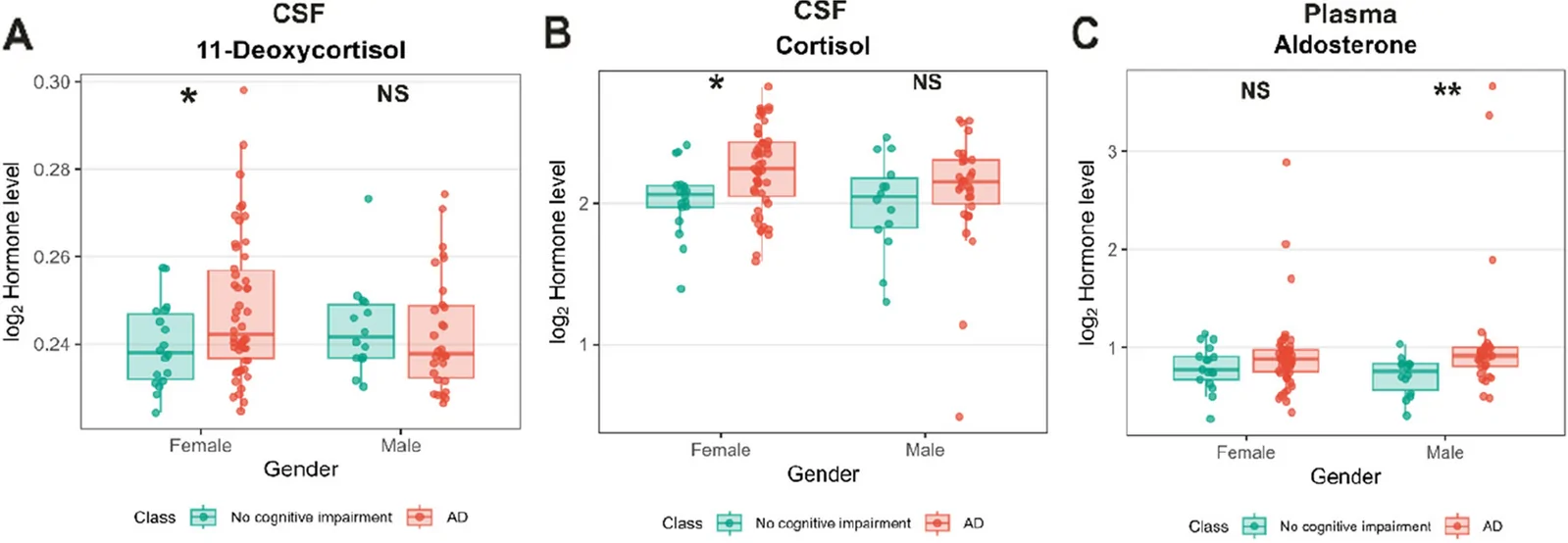
Hormones associated with AD**.** Boxplots showing log₂-transformed hormone levels stratified by gender and between no cognitive impairment and AD. Three representative hormones are shown: (**A**) CSF 11-deoxycortisol, (**B**) CSF cortisol, and (**C**) plasma aldosterone. Box plots show median values, with box edges representing the interquartile range (IQR; 25th–75th percentiles) and whiskers extending to 1.5 × IQR. Individual points indicate participants. Significance levels are indicated above each comparison: * *p* < 0.05, ** *p* < 0.01, and NS, not significant

Plasma hormone analyses revealed gender-specific patterns (Fig. 2). In female participants, lower plasma progesterone showed a significant association with AD (FC (95% CI) = 0.90, (0.81–0.99), p-value = 0.04). In male participants, elevated plasma 17-hydroxyprogesterone was associated with MCI (FC (95% CI) = 1.08 (1.00–1.15), p-value = 0.04), but not with AD or VaD. Elevated plasma estrone was associated with VaD in male participants (FC (95% CI) = 1.45 (1.05–1.99), p-value = 0.03). Additionally, higher plasma aldosterone was associated with AD in male participants (FC (95% CI) = 1.19 (1.06–1.33), p-value = 2.81e-03) (Fig. 3C), this was the only finding that remained significant after FDR correction (q = 0.02). The distribution of the non-significantly changed gender stratified hormones between NCI and AD in both plasma and CSF is displayed in Supplementary Fig. 1. Post-hoc power calculations indicated adequate power for the CSF cortisol finding in female participants (Cohen's d = 0.876, achieved power = 0.883). For plasma aldosterone in male participants, achieved power was moderate (Cohen's d = 0.791, achieved power = 0.666), reflecting the limited male NCI reference group (*n* = 14).

Gender-adjusted GLMs were also conducted. CSF cortisol was significantly elevated in individuals with AD compared to NCI (FC (95% CI) = 1.50 (1.03–2.18), *p*-value = 0.03; Supplementary Fig. 2). Plasma progesterone was significantly lower across all cognitive impairment groups compared to controls: MCI (FC (95% CI) = 0.88 (0.78–0.98), *p*-value = 0.02), AD (FC (95% CI) = 0.89 (0.82–0.96), *p*-value = 2.01e-03, q = 0.02), and VaD (FC (95% CI) = 0.9 (0.82–0.99), *p*-value = 0.03; Supplementary Fig. 3). Furthermore, plasma 17-hydroxyprogesterone was significantly decreased in individuals with VaD compared to controls (FC (95% CI) = 0.91 (0.83–1.00), *p*-value = 0.04).

### Hormone correlations

**Fig. 4:**
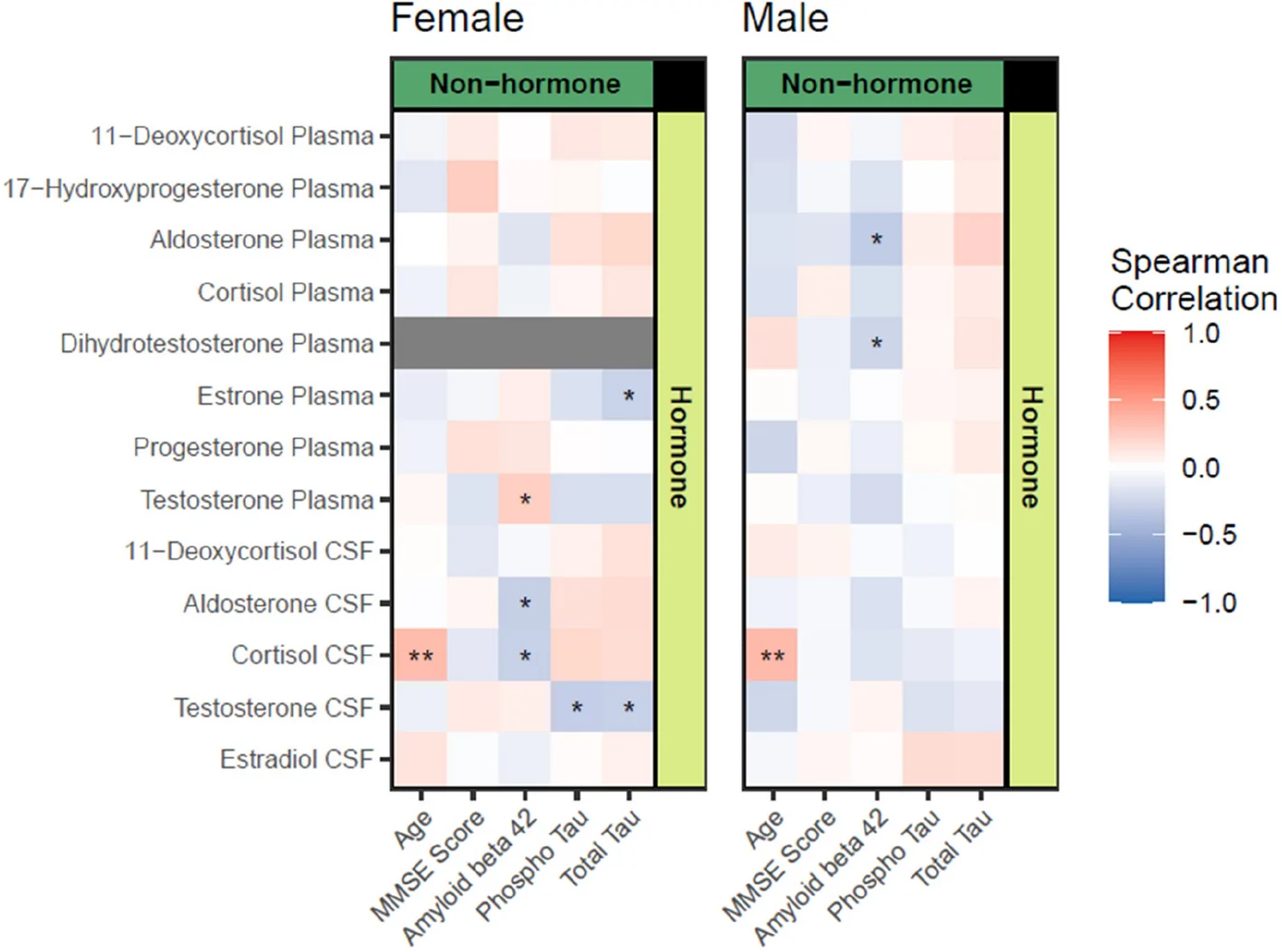
Correlation Matrix. Correlation Matrix: Heatmap of Spearman’s correlations between hormones and markers associated with AD. * indicate a q-value > 0.05, ** a q-value > 0.01 and *** a q-value > 0.001. q-values are obtained through multiple testing correction with FDR

Spearman correlation analyses revealed correlations between hormones, AD biomarkers, and cognitive function, with notable gender-specific patterns (Fig. 4, Supplementary Figs. 4 and 5).

CSF cortisol increased with age in both genders (female: *r* = 0.34, q = 0.01; male: *r* = 0.35, q = 1.9e-03; Supplementary Data Sheet 2), whereas plasma cortisol showed no age association. In female participants, higher CSF cortisol was associated with lower Aβ−42 (*r* = − 0.29, q = 0.01) and higher P-tau (*r* = 0.20, *p* = 0.04), indicative of AD pathology. CSF cortisol in female participants also correlated positively with aldosterone in CSF and plasma, multiple plasma steroids (progesterone, testosterone, cortisol), and CSF 11-deoxycortisol. In male participants, CSF cortisol correlated with CSF and plasma aldosterone, plasma estrone, and CSF 11-deoxycortisol.

Aldosterone showed gender-specific associations with AD biomarkers: CSF aldosterone correlated negatively with Aβ−42 in female participants, whereas plasma aldosterone showed this association in male participants. Plasma aldosterone correlated positively with T-tau in male but not female participants.

Testosterone displayed divergent gender-specific patterns. In female participants, CSF and plasma testosterone were negatively associated with P-tau and T-tau and positively associated with Aβ−42. In contrast, plasma testosterone in male participants correlated negatively with Aβ−42.

MMSE scores showed expected correlations with AD biomarkers, including positive associations with Aβ−42 and negative associations with T-tau. However, significant correlations with hormones were limited, with only plasma 17-hydroxyprogesterone showing a relationship with MMSE in female participants (*r* = 0.25, *p*-value = 0.01). Inter-hormone correlation patterns varied substantially by hormone class. Hormones detected and measured in both biofluids, including aldosterone, cortisol, 11-deoxycortisol, and testosterone, demonstrated strong correlations between their plasma and CSF concentrations. In contrast, estrogens showed minimal interconnectivity, plasma estrone and CSF estradiol correlated only with each other (negatively). At the opposite extreme, plasma progesterone and plasma 17-hydroxyprogesterone demonstrated the broadest correlation patterns, associating with all other plasma hormones except plasma estrone.

Exploratory analyses using dimensionality reduction methods did not show any separation of the dementia subgroups in the unsupervised PCA (Supplementary Fig. 6), nor for the supervised PLS-DA, yet the hormones with the most power to separate the dementia subgroups in CSF were cortisol, 11-deoxycortisol and aldosterone in both male and female subsets. For plasma hormones the best separation power came from progesterone, 17-hydroxyprogesterone and testosterone for female participants and from 11-deoxycortisol, progesterone and 17-hydroxyprogesterone for male participants, showing some consistency across the genders.

### Cortisol validation

**Fig. 5:**
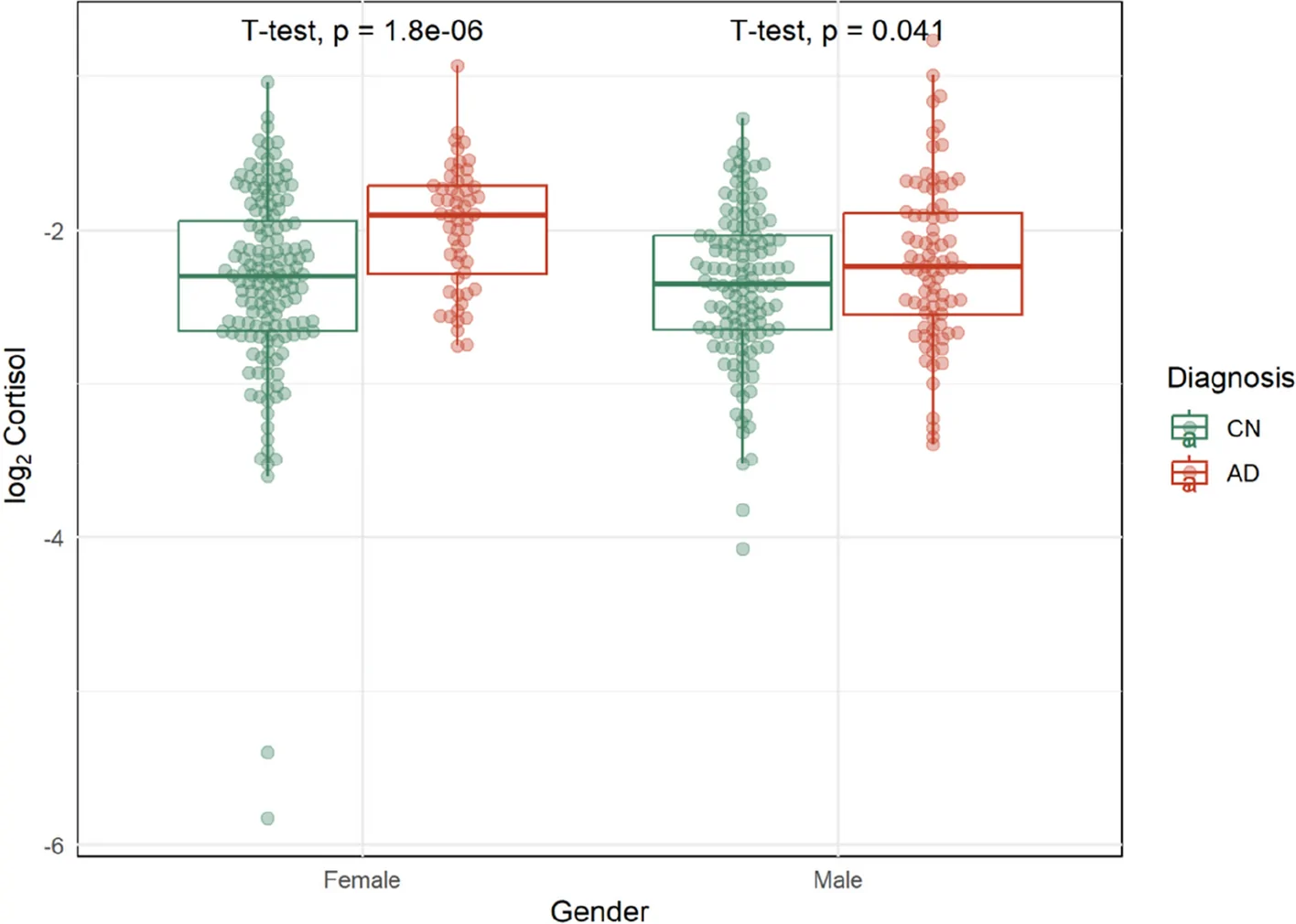
Differences between cortisol in ADNI. Boxplot with t-test comparison showing levels of cortisol in females and males with individuals that are cognitively healthy (CN) or have Alzheimer’s disease (AD)

Comparison of plasma cortisol measurements of 426 individuals from the ADNI cohort was carried out as an external validation. The cohort consisted of 153 cognitively healthy female participants, 133 cognitively healthy males, 56 female participants with AD and 84 male participants with AD. Participant characteristics and descriptive statistics for the ADNI validation cohort are provided in Supplementary Table 2. The mean normalized cortisol levels cognitively healthy female participants were 0.21, compared to 0.26 of the female participants with AD (log_2_ FC = 1.24, *p*-value = 1.79e-06, Fig. 5, Supplementary Data Sheet 3). The difference in male participants was less striking yet still significant, healthy male participants had a mean normalized cortisol level at 0.21 compared to male participants with AD 0.21 (log_2_ FC = 1.12, *p*-value = 0.04).

## Discussion

Here we have quantitatively measured hormone levels in both blood and CSF among older adults, addressing an important knowledge gap, particularly given the potential clinical implications for neurodegenerative diseases. For this we developed a method to quantify nine steroid hormones using mass spectrometry from CSF and plasma in a cohort of individuals in their 60 s and 70 s evaluated and diagnosed at the memory clinic at Rigshospitalet in Copenhagen. Our findings show significant gender specific associations between hormones and cognitive impairment specifically for CSF cortisol, plasma aldosterone and plasma progesterone.

### CSF Cortisol and its precursor were higher in female participants with AD

This study is not only one of the few to measure cortisol in CSF but the only one to assess it both in CSF and plasma simultaneously. CSF cortisol and its precursor CSF 11-deoxycortisol were significantly higher in female participants with AD (Fig. 1), while no significant alterations were found in plasma or in male participants. A weaker trend toward higher CSF cortisol was observed in vascular dementia for both genders. These results align with previous reports showing elevated plasma and CSF cortisol in AD and cognitive impairment [16–20]. Additional testing of plasma cortisol from the ADNI cohort also showed increased levels of cortisol in people with AD compared to cognitively healthy individuals, this difference being larger in the female population (Fig. 5). The reason we did not find any difference for plasma cortisol is likely due to the smaller sample size (n = 69 female vs. 203 female participants in ADNI, 44 male vs. 217 male participants in ADNI). Increased plasma cortisol in AD has previously been shown in ADNI [25, 26], but here we highlight the difference between female and male participants.

While the lack of a plasma effect (FC (95% CI) = 1.01 (0.96–1.06), *p*-value = 0.7 in female participants) may reflect limited statistical power, it suggests that CSF cortisol (FC (95% CI) = 1.13 (1.01–1.27), *p*-value = 0.04) is a more sensitive marker of cognitive impairment than plasma cortisol. This is not surprising, as plasma cortisol is more readily influenced by circadian variation and external exposures, whereas CSF cortisol more directly reflects central hypothalamic–pituitary–adrenal (HPA) axis activity.

Previous studies, such as the landmark study by Lupien et al., showed that elderly individuals with prolonged cortisol elevations over five years had reduced hippocampal volumes and memory deficits, with the degree of atrophy correlating strongly with cortisol levels [27]. Elevated CSF cortisol was observed only in female participants, which may partly reflect limited statistical power (18 NCI and 51 female particpants with AD vs. 14 NCI and 30 male participants with AD). However, there was little indication of a similar trend in male participants with AD, although a slight increase was observed in male participants with VaD. In female participants, CSF cortisol correlated negatively with Aβ−42 and positively with P-tau, suggestive of AD pathology, correlations that were not seen in male participants (Fig. 4), AD is known to present distinct molecular manifestations in men and women [28, 29]. It has been suggested that estradiol decline after menopause exacerbates glucocorticoid sensitivity, leading to higher central cortisol exposure in women [30], however, we saw no correlation between estradiol and cortisol in this study.

The concurrent elevation of CSF cortisol and its immediate precursor CSF 11-deoxycortisol, in the absence of corresponding changes in plasma 17-hydroxyprogesterone and with decreased plasma progesterone, points to compartment-specific alterations in steroidogenesis within the brain/CSF. Since 11-deoxycortisol is converted to cortisol by 11β-hydroxylase, these findings suggest upstream local synthesis, upregulated enzymatic activity, or reduced clearance of cortisol precursors in the brain rather than systemic overproduction. Such central amplification could reflect neuroinflammatory modulation of steroidogenic enzymes or glial-driven neurosteroidogenesis, processes known to be altered in AD, particularly in women [31]. Overall, our data highlight the potential of CSF cortisol and CSF 11-deoxycortisol as a mechanism to study further in AD, especially among the female population.

### Aldosterone linked to brain pathology in male participants

We observed higher plasma aldosterone levels in male participants with AD, with a similar but non-significant trend in female participants (Fig. 2), while CSF aldosterone showed no association. In men, plasma aldosterone correlated negatively with Aβ−42 and positively with T-tau, suggesting a possible link between peripheral mineralocorticoid activity and AD pathology (Fig. 4). Previous studies have implicated dysregulation of the renin–angiotensin–aldosterone system (RAAS) in both AD and vascular dementia [22, 32], and excessive aldosterone has been associated with cognitive impairment and reduced cerebral perfusion [21, 33]. Together, these findings suggest that elevated plasma aldosterone may contribute to neurodegeneration indirectly through vascular or inflammatory pathways rather than direct central accumulation, consistent with the lack of association in CSF. The stronger effect in male participants may reflect sex differences in RAAS regulation or vascular susceptibility.

### Progesterone, estrogens and testosterone

Plasma progesterone concentrations were significantly reduced in female participants with AD (Fig. 2), as well as across all disease categories (MCI-AD, AD, and VaD) in our combined models (Supplementary Fig. 3). Plasma 17-hydroxyprogesterone showed a similar but less pronounced decrease, it was reduced significantly in vascular dementia suggesting a shift towards mineralocorticoid synthesis.

Progesterone receptors are broadly expressed throughout the brain, in key cognitive regions including the hippocampus and frontal cortex, with no apparent restriction to specific cell types or hormonal status [34]. Progesterone also exerts important protective effects on the cardiovascular system that could be relevant to vascular health. The natural hormone acts as a vasodilator through modulation of calcium channels and enhancement of nitric oxide bioavailability, improving cerebral blood flow [35, 36].

While progesterone supplementation could represent a potential therapeutic strategy, implementing such treatment is far from straightforward. Progesterone can be found as different formulations, as an example, progestins, a range of progesterone derivatives commonly used for birth control and in hormone replacement therapy (HRT), may act differently than endogenous progesterone [37, 38]. Some studies have also reported higher dementia risk associated with HRT use, highlighting that we need more studies [39, 40]. The specific molecular composition of HRT formulations would be a critical factor in any prevention trials together with choosing the appropriate therapeutic window and dose.

A meta-analysis by Rocca et al. demonstrated that HRT may be detrimental for women in late post-menopause, specifically the age of the participant in our study (65–79), while conferring neuroprotective effects in early post-menopause (ages 50–60) [41]. This time-dependent effect has been confirmed in subsequent studies [7, 8, 42]. One study found HRT improved memory delay and was associated with larger brain volumes but only in women carrying the APOE4 genotype [43].

We observed decreased CSF estradiol in female participants with VaD (FC (95% CI) = 0.86 (0.74–0.98), *p*-value = 0.03), which interestingly was not seen in the AD group (FC (95% CI) = 1.03 (0.93–1.14), *p*-value = 0.6). In plasma, higher estrone was associated with VaD in male participants and showed a similar trend in MCI-AD and AD. These findings support gender-specific alterations in estrogenic pathways across dementias. Reduced CSF estradiol in female participants could contribute to vascular vulnerability, consistent with epidemiological studies linking shorter lifetime estrogen exposure or early menopause to greater dementia risk [5, 6]. One study of the UK Biobank found no causal link between estradiol exposure and brain health, suggesting that associations between estradiol and brain health is governed by hormone fluctuations [44]. Estrogens exert neuroprotective effects through modulation of synaptic plasticity, cerebral blood flow, and amyloid metabolism [45]. In men, however, elevated plasma estrone may reflect compensatory aromatization of testosterone under metabolic or vascular stress, possibly signalling altered peripheral-to-central steroid dynamics rather than direct neuroprotection.

No overall association was found between testosterone and cognitive decline, but gender-specific patterns emerged. In female participants, CSF and plasma testosterone correlated negatively with tau markers and positively with Aβ−42, suggesting a protective role. In male participants, plasma testosterone correlated negatively with Aβ−42, consistent with prior studies linking low testosterone to higher dementia risk [14, 15, 46]. These divergent associations likely reflect sex differences in androgen metabolism and receptor sensitivity, emphasizing that hormonal ratio balance, rather than absolute levels, may be key to maintaining cognitive health.

### Strengths and limitations

To our best knowledge this is the first comprehensive study to fully quantify aldosterone, 11-deoxycortisol, cortisol, and testosterone in both plasma and CSF, with a total of nine unique hormones across the two biofluids, from the same elderly individuals across diagnosis. Five distinct dementia subgroups were defined, based on a thorough clinical evaluation in the memory clinic. A limitation of this study is its cross-sectional design, with hormone concentrations measured at a single time point. Therefore, we cannot capture longitudinal hormone dynamics or determine whether the observed associations precede, accompany, or follow cognitive decline. These associations may reflect risk-related endocrine changes, compensatory responses, disease-related physiological alterations, or downstream consequences of neurodegeneration. Longitudinal studies with repeated hormone measurements and cognitive follow-up are needed to validate these findings and clarify the temporal relationship between steroid hormone dysregulation and dementia progression. As the persons in this study are considered vulnerable, sample collection did not follow a fasting period. This may have contributed to some measurement variability, particularly for hormones sensitive to sampling conditions. In addition, CSF cortisol exhibits diurnal variation, and lumbar puncture timing was not standardized across participants, which may have introduced further variability. Therefore, our findings require further validation in cohorts with standardized sampling protocols. Additionally, the stratification of participants by both dementia subtype and gender resulted in relatively small group sizes, which may have limited our statistical power. Post-hoc power calculations confirmed adequate power for the cortisol finding in female participants, while power for the aldosterone finding in male participants was moderate (0.666), primarily constrained by the small NCI male reference group. Notably, the aldosterone finding remained significant after FDR correction despite this limitation, suggesting the underlying effect size is robust. Nevertheless, larger sex-stratified studies are warranted to confirm these findings.

## Conclusion

This study demonstrates distinct, gender-specific associations between steroid hormones and cognitive impairment. Female participants showed elevated CSF cortisol and reduced plasma progesterone, whereas male participants showed higher plasma aldosterone. These findings suggest that dysregulation of glucocorticoid, mineralocorticoid, and sex-steroid pathways could contribute to neurodegeneration through both central and vascular mechanisms. The divergent hormone patterns underscore the need for sex-specific considerations in AD and related dementias research.

## Supplementary Information

Supplementary Material 1.

## Data Availability

The dataset analyzed here is not directly publicly available, for the privacy of the participants, in compliance with EU and Danish data protection law. The data can be accessed upon reasonable request and relevant legal permission from the danish data protection agency. Data access requests should be directed to S.G.H..

## Abbreviations

AD: Alzheimer’s disease
ADNI: Alzheimer’s Disease Neuroimaging Initiative
APOE ε4: Apolipoprotein E ε4
Aβ-42: Amyloid-β 42
CI: Confidence interval
CN: Cognitively healthy control
CSF: Cerebrospinal fluid
dMRM: Dynamic multiple reaction monitoring
FC: Fold change
GLM: Generalized linear model
HRT: Hormone replacement therapy
LC–MS: Liquid chromatography–tandem mass spectrometry
MCI: Mild cognitive impairment
NCI: No cognitive impairment
PCA: Principal component analysis
PLS-DA: Partial least squares–discriminant analysis
P-tau: Phosphorylated tau
RAAS: Renin–angiotensin–aldosterone system
SEM: Standard error of the mean
T-tau: Total tau
VIP: Variable importance in projection

## References

[1] Rajan KB, Weuve J, Barnes LL, McAninch EA, Wilson RS, Evans DA. Population estimate of people with clinical Alzheimer’s disease and mild cognitive impairment in the United States (2020–2060). Alzheimers Dement. 2021;17:1966–75. 10.1002/alz.12362. PMC9013315 34043283

[2] Matthews FE, Stephan BCM, Robinson L, Jagger C, Barnes LE, Arthur A, et al. A two decade dementia incidence comparison from the cognitive function and ageing studies i and II. Nat Commun. 2016. 10.1038/ncomms11398. PMC4838896 27092707

[3] Beam CR, Kaneshiro C, Jang JY, Reynolds CA, Pedersen NL, Gatz M. Differences between women and men in incidence rates of dementia and Alzheimer’s Disease. J Alzheimer’s Dis. 2018;64:1077–83. 10.3233/JAD-180141. PMC6226313 30010124

[4] Zhao Y, Wang Q, Fu C, Li M, Hao W, Wang X, et al. Sex hormones and risk of incident dementia in men and postmenopausal women. Clin Endocrinol (Oxf). 2025;103:366–75. 10.1111/cen.15271. 40350794

[5] Id JG, Harris K, Peters SAE, Id MW, Gong J, Harris K, et al. Reproductive factors and the risk of incident dementia: a cohort study of UK Biobank participants. PLoS Med. 2022;18:1–23. 10.1371/journal.pmed.1003955. PMC8982865 35381014

[6] Liao H, Cheng J, Pan D, Deng Z, Liu Y, Jiang J, et al. Association of earlier age at menopause with risk of incident dementia, brain structural indices and the potential mediators: a prospective community-based cohort study. EClinicalMedicine. 2023;60:1–14. 10.1016/j.eclinm.2023.102033. PMC10314163 37396803

[7] Matyi JM, Rattinger GB, Schwartz S, Buhusi M, Tschanz JT. Lifetime estrogen exposure and cognition in late life: the Cache County Study. Menopause J OfThe North Am Menopause Soc. 2019;26:1366–74. 10.1097/GME.0000000000001405. PMC7448538 31613825

[8] Coughlan GT, Betthauser TJ, Boyle R, Koscik RL, Klinger HM, Chibnik LB, et al. Association of age at menopause and hormone therapy use with Tau and β -amyloid positron emission tomography. JAMA Neurol. 2023;80:462–73. 10.1001/jamaneurol.2023.0455. PMC10071399 37010830

[9] Park HK, Sc M, Ph D, Marston L, Sc B, Sc M, et al. The effects of estrogen on the risk of developing dementia: a cohort study using the UK biobank data. Am J Geriatr Psychiatry. 2024;32:1–14. 10.1016/j.jagp.2024.01.025. 38310026

[10] Hao W, Fu C, Dong C, Zhou C, Sun H, Xie Z, et al. Age at menopause and all-cause and cause-specific dementia: a prospective analysis of the UK Biobank cohort. Hum Reprod. 2023;38:1746–54. 10.1093/humrep/dead130 PMC10663050 37344154

[11] De LAG, Barth C, Maximov II, Van Der MD, Kaufmann T, Agartz I, et al. Women’s brain aging: Effects of sex-hormone exposure, pregnancies, and genetic risk for Alzheimer’s disease. Hum Brain Mapp. 2020;41:5141–50. 10.1002/hbm.25180. PMC7670641 32856754

[12] Najar J, Hallstrom T, Zettergren A, Johansson L, Joas E, Fa MM, et al. Reproductive period and preclinical cerebrospinal fluid markers for Alzheimer disease: a 25-year study. Menopause J OfThe North Am Menopause Soc. 2021;28:1099–107. 10.1097/GME.0000000000001816. PMC8462446 34225325

[13] Zhang Z, Kang D, Li H. Testosterone and cognitive impairment or dementia in middle-aged or aging males : causation and intervention, a systematic review and meta-analysis. J Geriatr Psychiatry Neurol. 2021;34:405–17. 10.1177/0891988720933351. 32602403

[14] Marriott RJ, Murray K, Flicker L, Hankey GJ, Matsumoto AM, Dwivedi G, et al. Lower serum testosterone concentrations are associated with a higher incidence of dementia in men: The UK Biobank prospective cohort study. Alzheimer’s Dement. 2022;18:1907–18. 10.1002/alz.12529. 34978125

[15] Yeung CHC, Yeung SLA, Kwok MK, Zhao JV, Schooling CM. The influence of growth and sex hormones on risk of alzheimer ’ s disease : a mendelian randomization study. Neuro-Epidemiology. 2023;38:745–55. 10.1007/s10654-023-01015-2 37253999

[16] Peña-bautista C, Baquero M, Ferrer I, Hervás D, Vento M, García-blanco A. Neuropsychological assessment and cortisol levels in biofluids from early Alzheimer’s disease patients. Exp Gerontol. 2019;123:10–6. 10.1016/j.exger.2019.05.007. 31117002

[17] Zheng B, Tal R, Yang Z, Middleton L, Udeh-momoh C. Cortisol hypersecretion and the risk of Alzheimer’s disease: a systematic review and meta-analysis. Ageing Res Rev. 2020. 10.1016/j.arr.2020.101171. 32971258

[18] Ennis GE, Resnick SM, Ferrucci L, Brien RJO. Long-term cortisol measures predict Alzheimer disease risk. Neurology. 2016;88:371–8. 10.1212/WNL.0000000000003537. PMC5272965 27986873

[19] Pietrzak RH, Laws SM, Lim YY, Bender SJ, Porter T, Doecke J, et al. Archival report plasma cortisol, brain amyloid-β, and cognitive decline in preclinical Alzheimer’s disease: a 6-year prospective cohort study. Biol Psychiatry Cogn Neurosci Neuroimaging. 2017;2:45–52. 10.1016/j.bpsc.2016.08.006. 29560886

[20] Udeh-momoh CT, Su B, Evans S, Zheng B, Sindi S, Sciences H. Cortisol, amyloid-β, and reserve predicts Alzheimer’s disease progression for cognitively normal older adults. J Alzheimer’s Dis. 2019;70:553–62. 10.3233/JAD-181030. 31256117

[21] Yagi S, Akaike M, Aihara K, Iwase T, Yoshida S, Sumitomo-ueda Y, et al. High plasma aldosterone concentration is a novel risk factor of cognitive impairment in patients with hypertension. Hypertens Res. 2011. 10.1038/hr.2010.179. 20861841

[22] Hong N, Kim KJ, Yu MH, Jeong SH, Lee S, Lim JS. Risk of dementia in primary aldosteronism compared with essential hypertension : a nationwide cohort study. Alzheimers Res Ther. 2023;15:1–12. 10.1186/s13195-023-01274-x. PMC10416485 37568223

[23] Hasselbalch SG, Andersen BB, Ejlerskov P, Frederiksen KS, Nielsen JE, Nielsen TR, et al. The Danish dementia research centre: integrating patient care, clinical research, and national educational services. J Alzheimer’s Dis. 2025;107:883–98. 10.1177/13872877251365195. 40776623

[24] Mueller SG, Weiner MW, Thal LJ, Petersen RC, Jack CR, Jagust W, et al. Ways toward an early diagnosis in Alzheimer’s disease: The Alzheimer’s Disease Neuroimaging Initiative (ADNI). Alzheimer’s Dement. 2005;1:55–66. 10.1016/j.jalz.2005.06.003. PMC1864941 17476317

[25] Toledo JB, Weiner MW, Jack CR. Cardiovascular risk factors, cortisol, and amyloid-b deposition in Alzheimer’s Disease Neuroimaging Initiative. Alzheimer’s Dement. 2012;8:483–9. 10.1016/j.jalz.2011.08.008. PMC3668456 23102118

[26] Borkowski K, Yin C, Kindt A, Liang N, Blach C, Newman JW, et al. Metabolic Alteration in Oxylipins and Endocannabinoids Point to an Important Role for Soluble Epoxide Hydrolase and Inflammation in Alzheimer’s Disease - Finding from Alzheimer’s Disease Neuroimaging. BioRxiv 2025:1–28. 10.1186/s13195-025-01939-9 PMC12857118 41495790

[27] Lupien SJ, Leon MD, Santi SD, Convit A, Tarshish C, Thakur M, et al. Cortisol levels during human aging predict hippocampal atrophy and memory deficits. Nat Neurosci. 1998;1:69–73. 10.1038/271 10195112

[28] Xu J, Bankov G, Kim M, Wretlind A, Lord J, Green R, et al. Integrated lipidomics and proteomics network analysis highlights lipid and immunity pathways associated with Alzheimer’s disease. Transl Neurodegener. 2020;9:1–15. 10.1186/s40035-020-00215-0 PMC7504646 32951606

[29] Wretlind A, Xu J, Chen W, Velayudhan L, Ashton NJ, Zetterberg H, et al. Lipid profiling reveals unsaturated lipid reduction in women with Alzheimer’s disease. Alzheimers Dement. 2025;21:1–13. 10.1002/alz.70512. PMC12365783 40832908

[30] Cohn AY, Grant LK, Nathan MD, Wiley A, Abramson M, Harder JA, et al. Effects of sleep fragmentation and estradiol decline on cortisol in a human experimental model of menopause. J Clin Endocrinol Metab. 2023;108:1347–57. 10.1210/clinem/dgad285. PMC10584010 37207451

[31] Klyubin I, Ondrejcak T, Hu N, Rowan MJ. ScienceDirect Glucocorticoids, synaptic plasticity and Alzheimer ’ s disease. Curr Opin Endocr Metab Res. 2022;25:100365. 10.1016/j.coemr.2022.100365.

[32] Tayler HM, Kehoe PG, Miners JS. Dysregulation of the renin-angiotensin system in vascular dementia. Brain Pathol. 2024;34:1–12. 10.1111/bpa.13251. PMC11189771 38454306

[33] Hajjar I, Hart M, Mack W, Lipsitz LA. Aldosterone, cognitive function, and cerebral hemodynamics in hypertension and antihypertensive therapy. Am J Hypertens. 2015;28:319–25. 10.1093/ajh/hpu161. PMC4402350 25213687

[34] Brinton RD, Thompson RF, Foy MR, Baudry M, Wang J, Finch CE, et al. Progesterone receptors : form and function in brain. Front Neuroendocrinol. 2008;29:313–39. 10.1016/j.yfrne.2008.02.001. PMC2398769 18374402

[35] Oelkers WKH. Effects of estrogens and progestogens on the renin-aldosterone system and blood pressure. Steroids. 1996. 10.1016/0039-128X(96)00007-4. 8732994

[36] Hermenegildo C, Oviedo PJ, Garcia-Martinez MC, Garcia-Perez MA, Tarin JJ, Cano A. Progestogens stimulate prostacyclin production by human endothelial cells. Hum Reprod. 2005;20:1554–61. 10.1093/humrep/deh803. 15734756

[37] Cordina-duverger E, Truong T, Anger A, Sanchez M, Arveux P, Kerbrat P, et al. Risk of breast cancer by type of menopausal hormone therapy: a case-control study among post-menopausal women in France. PLoS ONE. 2013;8:1–9. 10.1371/journal.pone.0078016. PMC3815310 24223752

[38] Asi N, Mohammed K, Haydour Q, Gionfriddo MR, Vargas OLM, Prokop LJ, et al. Progesterone vs. synthetic progestins and the risk of breast cancer: a systematic review and meta-analysis. Syst Rev. 2016;5:1–8. 10.1186/s13643-016-0294-5. PMC4960754 27456847

[39] Savolainen-peltonen H, Rahkola-soisalo P, Hoti F, Vattulainen P, Gissler M, Ylikorkala O, et al. Use of postmenopausal hormone therapy and risk of Alzheimer’s disease in Finland: nationwide case-control study. BMJ. 2019;364:1–8. 10.1136/bmj.l665. PMC6402043 30842086

[40] Pourhadi N, Mørch LS, Holm EA, Torp-pedersen C, Meaidi A. Menopausal hormone therapy and dementia: nationwide, nested case-control study. BMJ. 2023. 10.1136/bmj-2022-072770. PMC10302215 37380194

[41] Rocca WA, Grossardt BR, Shuster LT. Molecular and Cellular Endocrinology Oophorectomy, estrogen, and dementia: A 2014 update. Mol Cell Endocrinol. 2014;389:7–12. 10.1016/j.mce.2014.01.020. PMC4040304 24508665

[42] Song Y, Li S, Li X, Chen X, Wei Z, Liu Q, et al. The effect of estrogen replacement therapy on Alzheimer’s disease and parkinson’s disease in postmenopausal women: a meta-analysis. Front Neurosci. 2020;14:1–13. 10.3389/fnins.2020.00157. PMC7076111 32210745

[43] Saleh RNM, Hornberger M, Ritchie CW, Minihane AM. Hormone replacement therapy is associated with improved cognition and larger brain volumes in at-risk APOE4 women: results from the European Prevention of Alzheimer’s Disease (EPAD) cohort. Alzheimer’s Res Ther. 2023;15:1–13. 10.1186/s13195-022-01121-5. PMC9830747 36624497

[44] Oppenheimer H, van der Meer D, Schindler LS, Crestol A, Shadrin A, Andreassen OA, et al. No causal links between estradiol and female’s brain and mental health using Mendelian randomization. Nat Commun. 2025;16:1–13. 10.1038/s41467-025-65878-7 PMC12680690 41350255

[45] Kim G, Park K, Jeong G. Effects of Sex Hormones and Age on Brain Volume in Post-Menopausal Women. J Sex Med. 2018;15:662–70. 10.1016/j.jsxm.2018.03.006. 29628218

[46] Liu L, Zhang C, Lv X, Lai X, Xu L, Feng J, et al. Sex-specific associations between lipids and cognitive decline in the middle-aged and elderly: a cohort study of Chinese adults. Alzheimer’s Res Ther. 2020;12:1–13. 10.1186/s13195-020-00731-1. PMC7722300 33287901

